# 36-month durability of ultrasound renal denervation for hypertension resistant to combination therapy in RADIANCE-HTN TRIO

**DOI:** 10.1038/s41440-024-01854-w

**Published:** 2024-09-27

**Authors:** Michael J. Bloch, Ajay J. Kirtane, Michel Azizi, Felix Mahfoud, Jan Basile, Joost Daemen, Manish Saxena, Lisa Thackeray, Maureen McGuire, Lisa Claude, Roland E. Schmieder, On behalf of the RADIANCE-HTN Investigators, Yale Wang, Nedaa Skeik, Richard Bae, Amy McMeans, JoAnne Goldman, Rose Peterson, James Stephen Jenkins, Isabelle Tutor, Michael Harrison, Angel Penning, Chandan Devireddy, Janice Lea, Amanda Fiebach, Claudia Merlin, Florian Rader, Suhail Dohad, Anne Tran, Kirin Bhatia, Naomi D. L. Fisher, Piotr Sobieszczyk, Ian Halliday, Tay Munson, Jason Lindsey, Steven Laster, Mathew Bunte, Anthony Hart, Dana King, Jamie Hall, Kintur Sanghvi, Courtney Krathen, Luot Lewis, Ashley Willitts, Thomas Todoran, Jan Basile, Anthony Awkar, Casey Palmer, Anna Tecklenburg, John Schindler, John Pacella, Matthew Muldoon, MaryJo Albright, Tracy Nicholson, John Flack, Youseff Chami, Abdul Moiz Hafiz, Emily Starkey, Kristal Adams, Nelson Bernardo, Judith Veis, Hayder Hashim, Suman Singh, Donna Whitman, Rick Stouffer, Alan Hinderliter, Meghan Allen, Tatum Scholl, Pete Fong, James Gainer, Sherron Crook, Ellen Hatchcock, Debbie Cohen, Jay Giri, Taisei Kobayashi, Robin Neubauer, Suveeksha Naidu, Ajay J. Kirtane, Jai Radhakrishnan, Candido Batres, Suzanne Edwards, Matheen Khuddus, Suzanne Zentko, Abby Touchton, Marti Roberson, Michael J. Bloch, Abhilash Akinapelli, Lisa English, Bridget Neumann, Farrel Mendelsohn, Hutton Brantley, Thomas Cawthon, Susan DeRamus, Wesley Wade, Robert Fishman, Edward Tuohy, Jessica LeBlanc, Tina McCurry, Amar Krishnaswamy, Luke Laffin, Christopher Bajzer, Marilyn Boros, Monica Branche, Josephine Abraham, Anu Abraham, Inge Stijleman, David Hsi, Scott Martin, Edward Portnay, Maryann Fiebach, Carolina Garavito, Todd Adams, Andrew Teklinski, Adam Leech, Patrick Drilling, Lynda Tulik, Keith Benzuly, James Paparello, Dan Fintel, Haydee Ramirez, Lauren Kats, Paul Huang, Santanu Biswas, Serena Risher, Kristina Pratt, Uzoma Ibebuogu, Karen Johnson, William Cushman, Lisa Jones, Leigh Jackson, David Landers, Tilak Pasala, Thomas Salazer, Peter Canino, Patricia Arakelian, Yi-Ming Yang, Asma Khaliq, Mitchell Weinberg, Yihenew Abetu, Alana Gulliver, J. P. Reilly, Joseph Garasic, Atul Chugh, Barry Bertolet, Brian Go, Raghava Gallapudi, Joel Cohn, Kevin Rogers, Manish Saxena, Anthony Mathur, Ajay Jain, Armida Balawon, Oliver Zongo, Christine Topham, Andrew Sharp, Richard Anderson, Elizabeth Thompson, Nikki Spiro, Elizabeth Hodges, Jaqueline Holder, Timothy Ellam, Alan Bagnall, Ralph Jackson, Victoria Bridgett, Peter Wilson, Neelanjan Das, Timothy Doulton, David Loader, Gemma Hector, Terry Levy, Clare Bent, Vivek Kodoth, Stephanie Horler, Sara Nix, Nicholas Robinson, Firas Al-Janabi, Jeremy Sayer, Sudha Ganesh Iyer, Emily Redman, Jonaifah Ramirez, Sandosh Padmanabhan, Faisal Sharif, Aishah Alhmoudi, Mattia Lunardi, Eileen Coen, Nicola Glynn, Felix Mahfoud, Lucas Lauder, Saarraaken Kulenthiran, Christina Koch, Angelika Wachter, Roland Schmieder, Axel Schmid, Dennis Kannenkeril, Ulrike Heinritz, Kerstin Endres-Frohlich, Philipp Lurz, Karl Rommel, Karl Fengler, Martin Petzold, Margit Büttner, Joachim Weil, Tolga Agdirlioglu, Tanja Köllner, Jeannine Stephan, Nikolaos Dagkonakis, Frank Hamann, Ute Ettl, Ulrike Petzsche, Peter Reimer, Martin Hausberg, Ralf Hinrichs, Isabella Di Ponio-Voit, Matthias Lutz, Philippe Gosse, Antoine Cremer, Panteleimon Papadopoulos, Julie Gaudissard, Florent Maire, Michel Azizi, Marc Sapoval, Marine Livrozet, Asma Regrag, Valerie Paquet, Pascal Delsart, Justin Hennicaux, Coralie Sommeville, Fabien Bertrand, Joost Daemen, Melvin Lafeber, Victor Zeijen, Amo Ruiter, Elisabeth Huijskens, Jan van Ramshorst, Panagiotis Xaplanteris, Rachid Briki, Quentin de Hemptinne, Severine Pascal, Katty Renard, Pascal Lefebvre, Bert Ferdinande, Juan F. Iglesias, Georg Ehert, Laetitia Gallego, Kevin Dobretz, Sylviane Bottone, Josh Costello, Courtney Krathan, Andrew McElvarr, John Reilly, Stephen Jenkins, Michael Cash, Shannon Williams, Maria Jarvis, Cheryl Laffer, Mark Robbins, Sarita Maddel, Maryanne Ducey, Suzanne Rose, Elizabeth DelMastro, Sripal Bangalore, Stephen Williams, Stanley Cabos, Carolina Rodriguez Alvarez, Eric Powers, Emily Hodskins, Vijay Paladugu, Bryan Wells, Hyun-Min Kim, Mohammad Rashid, Theophilus Owan, Iran Lavasani, Hailey Neilson, David Calhoun, Thomas McElderry, William Maddox, Suzanne Oparil, Sheila Kinder, Joseph Garasic, Doug Drachman, Randy Zusman, Kenneth Rosenfield, Danny Do, James O’Meara, Ilie Barb, Abby Foster, Alice Boyette, Desmond Jay, Robert Schwartz, Jo Anne Goldman, Jessie Goldman, Gary Ledley, Nancy Katof, Srinivasa Potluri, Scott Biedermann Jacquelyn Ward, Megan White, Laura Mauri, Piotr Sobieszczky, Alex Smith, Laura Aseltine, Eric Pauley, Tyrone Wade, David Zidar, Mehdi Shishehbor, Barry Effron, Marco Costa, Terence Semenec, Chanwit Roongsritong, Priscilla Nelson, Debbie Cohen, Jay Giri, Robin Neubauer, Thu Vo, Atul R. Chugh, Pei-Hsiu Huang, Powell Jose, Michael Jones, Manish Saxena, Melvin D. Lobo, Anthony Mathur, Ajay Jain, Armida Balawon, Olivier Zongo, Terry Levy, Clare Bent, David Beckett, Nicki Lakeman, Sarah Kennard, Andrew Sharp, Richard J. D’Souza, Sarah Statton, Lindsay Wilkes, Christine Anning, Jeremy Sayer, Sudha Ganesh Iyer, Nicholas Robinson, Annaliza Sevillano, Madelaine Ocampo, Robert Gerber, Mohamad Faris, Andrew John Marshall, Janet Sinclair, Hayley Pepper, Justin Davies, Neil Chapman, Paula Burak, Paula Carvelli, Sachin Jadhav, Jane Quinn, Lars Christian Rump, Johannes Stegbauer, Lars Schimmöller, Sebastian Potthoff, Claudia Schmid, Sylvia Roeder, Lukas Hafer, Felix Mahfoud, Michael Böhm, Sebastian Ewen, Saarraaken Kulenthiran, Angelika Wachter, Christina Koch, Karl Fengler, Karl-Philipp Rommel, Kai Trautmann, Roland E. Schmieder, Christian Ott, Axel Schmid, Michael Uder, Ulrike Heinritz, Kerstin Fröhlich-Endres, Sabine Genth-Zotz, Denise Kämpfner, Armin Grawe, Johannes Höhne, Bärbel Kaesberger, Constantin von zur Mühlen, Dennis Wolf, Markus Welzel, Gudrun Heinrichs, Barbara Trabitzsch, Hervé Trillaud, Erika Cornu, David Fouassier, Aurélien Lorthioir, Valérie Paquet, Atul Pathak, Benjamin Honton, Marianne Cottin, Frédéric Petit, Pierre Lantelme, Constance Berge, Pierre-Yves Courand, Fatou Langevin, Benjamin Longere, Guillaume Ledieu, François Pontana, Lida Feyz, Arno Ruiter, Elisabeth Huyskens, Peter Blankestijn, Michiel Voskuil, Zwaantina Rittersma, Helma Dolmans, A. A. Kroon, W. H. van Zwam, Jeannique Vranken, Claudia de Haan, Alexandre Persu, Jean Renkin, Frédéric Maes, Christophe Beauloye, Jean-Philippe Lengelé, Dominique Huyberechts, Anne Bouvier, Adam Witkowski, Andrzej Januszewicz, Jacek Kądziela, Aleksander Prejbisj, Dagmara Hering, Dariusz Ciecwierz, Milosz J. Jaguszewski, Radoslaw Owczuk, Ronald Victor, Courtney Walsh, Jonathan Williams, Sandeep Aggarwal, Scott Biedermann, Jeremy Sayer, Sudha Ganesh Iyer, Nicholas Robinson, Sadat Ali Edroos, Amit Patel, David Beckett, Justin Davies, Neil Chapman, Matthew Shun Shin, James Howard, Andrew S. P. Sharp, Anil Joseph, Richard D’Souza, Robert Gerber, Mohamad Faris, Andrew John Marshall, Cristina Elorz, Philipp Lurz, Robert Höllriegel, Karl Fengler, Karl-Philipp Rommel, Michael Böhm, Sebastian Ewen, Jelena Lucic, Roland E. Schmieder, Christian Ott, Michael Uder, Christian Rump, Johannes Stegbauer, Patric Kröpil

**Affiliations:** 1 Department of Medicine, University of Nevada School of Medicine, Vascular Care, Reno, NV USA; 2 Renown Regional Medical Center, Reno, NV USA; 3 Columbia University Medical Center/New York-Presbyterian Hospital and the Cardiovascular Research Foundation, New York, NY USA; 4 Université Paris Cité, F-75006 Paris, France; 5 AP-HP, Hôpital Européen Georges-Pompidou, Hypertension Department and DMU CARTE, F-75015 Paris, France; 6 INSERM, CIC1418, F-75015 Paris, France; 7 Chefarzt Klinik für Kardiologie, Universitäres Herzzentrum Basel, Universitätsspital Basel, Basel, Switzerland; 8 Institute for Medical Engineering and Science, Massachusetts Institute of Technology, Cambridge, MA USA; 9 Division of Cardiovascular Medicine, Medical University of South Carolina, Ralph H. Johnson VA Medical Center, Charleston, SC USA; 10 Erasmus MC, University Medical Center Rotterdam, Department of Cardiology, Rotterdam, NL The Netherlands; 11 Barts NIHR Biomedical Research Centre, William Harvey Research Institute, Queen Mary University of London, London, UK; 12 NAMSA, Minneapolis, MN USA; 13 Recor Medical Inc., Palo Alto, CA USA; 14 Nephrology and Hypertension, University Hospital Erlangen, Friedrich Alexander University, Erlangen, Germany; 15 Minneapolis Heart Institute Foundation, Minneapolis, MN USA; 16 Ochsner Heart and Vascular Institute, New Orleans, LA USA; 17 Emory University, Atlanta, GA USA; 18 Cedars-Sinai Medical Center, Los Angeles, CA USA; 19 The Brigham and Women’s Hospital, Boston, MA USA; 20 Saint Lukes Hospital of Kansas City, Kansas City, MO USA; 21 Deborah Heart & Lung Center, Brown Mills, NJ USA; 22 Medical University of South Carolina, Charleston, SC USA; 23 University of Pittsburgh Medical Center, Pittsburgh, PA USA; 24 Southern Illinois University School of Medicine, Springfield, IL USA; 25 MedStar Washington Health Research, Baltimore, MD USA; 26 The University of North Carolina, Chapel Hill, NC USA; 27 Vanderbilt University Medical Center, Nashville, TN USA; 28 Penn Medicine, Philadelphia, PA USA; 29 Columbia University Medical Center New York, New York, NY USA; 30 The Cardiac and Vascular Institute Gainesville, Gainesville, FL USA; 31 Cardiology PC, Birmingham, AL USA; 32 Bridgeport Hospital, Bridgeport, CT USA; 33 Cleveland Clinic Foundation, Cleveland, OH USA; 34 University of Utah Medical Center, Salt Lake City, UT USA; 35 Stamford Hospital, Stamford, CT USA; 36 Munson Medical Center, Traverse City, MI USA; 37 Northwestern University, Chicago, IL USA; 38 Swedish Medical Center, Seattle, WA USA; 39 University of Tennessee, Memphis, Memphis, TN USA; 40 Hackensack University, Hackensack, NJ USA; 41 Northwell Health- New York, New York, NY USA; 42 Stony Brook Medicine, Stony Brook, USA; 43 Massachusetts General Hospital, Boston, MA USA; 44 Franciscan Health, Indianapolis, IN USA; 45 Cardiology Associates of North Mississippi, Tupelo, MS USA; 46 WakeMed Clinical Research Institute, Raleigh, NC USA; 47 San Diego Cardiac Center, San Diego, CA USA; 48 Sparrow Clinical Research Institute, Lansing, USA; 49 University of Colorado, Denver, CO USA; 50 St Barts Health, London, UK; 51 University Hospital of Wales Cardiff, Cardiff, UK; 52 Freeman Hospital Newcastle, Newcastle upon Tyne, UK; 53 Kent and Canterbury Hospital, Canterbury, UK; 54 Royal Bournemouth Hospital, Bournemouth, UK; 55 Cardiothoracic Centre Basildon University Hospital Essex, Basildon, UK; 56 Queen Elizabeth University Hospital Glasgow, Glasgow, UK; 57 University Hospital Galway, Galway, Ireland; 58 University Clinic of Saarland- Homburg, Homburg, Germany; 59 University Clinic Erlangen, Erlangen, Germany; 60 Heart Center, Leipzig, Leipzig, Germany; 61 Sana Kliniken Lubeck GmbH, Lübeck, Germany; 62 Klinikum Konstanz, Konstanz, Germany; 63 Klinikum Karlsruhe, Karlsruhe, Germany; 64 UKSH Kiel, Kiel, Germany; 65 Hôpital Saint-André – CHU, Bordeaux, France; 66 Hôpital Européen Georges-Pompidou, Paris, France; 67 CHRU Lille, Lille, France; 68 Erasmus MC, Rotterdam, The Netherlands; 69 Noordwest Zienkenhuisgroeo – Alkmaar, Alkmaar, Netherlands; 70 UMC Saint Pierre, Bruxelles, Belgium; 71 Hopital Civel Marie Curie Charleroi, Charleroi, Belgium; 72 ZOL Genk, Genk, Belgium; 73 Hopitaux Universitaries Geneva, Geneva, Switzerland; 74 NYU Langone Medical Center, New York, NY USA; 75 University of Alabama, Birmingham, AL USA; 76 Mass General Hospital, Boston, MA USA; 77 Drexel University, Philadelphia, PA USA; 78 The Heart Hospital Baylor Plano, Plano, TX USA; 79 University Hospitals Cleveland Medical Center, Cleveland, OH USA; 80 University of Pennsylvania, Philadelphia, USA; 81 Sutter Medical Center, Sacramento, CA USA; 82 Baptist Health Lexington, Lexington, KY USA; 83 Barts Health NHS Trust, London, UK; 84 University Hospitals Dorset NHS Foundation Trust, Dorset, UK; 85 The Royal Devon and Exeter NHS Foundation Trust, Exeter, UK; 86 Mid and South Essex NHS Foundation Trust, Essex, UK; 87 East Sussex Healthcare NHS Trust, Sussex, UK; 88 Imperial College Healthcare NHS Trust, London, UK; 89 Nottingham University Hospitals NHS Trust, Nottingham, UK; 90 Düsseldorf University Hospital, Düsseldorf, Germany; 91 Saarland University Hospital, Homburg, Germany; 92 University Hospital Erlangen, Erlangen, Germany; 93 Katholisches Klinikum Mainz, Mainz, Germany; 94 Freiburg University and Faculty of Medicine, Freiburg, Germany; 95 Clinique Pasteur / GCVI, Toulouse, France; 96 Hôpital de la Croix Rousse, Lyon, France; 97 University Medical Center Utrecht, Utrecht, The Netherlands; 98 Maastricht University Hospital, Maastricht, The Netherlands; 99 Cliniques Universitaires Saint-Luc, Brussels, Belgium; 100 Institute of Cardiology, Warsaw, Poland; 101 Medical University of Gdansk, Gdansk, Poland; 102 The Essex Cardiothoracic Centre, Essex, UK; 103 Hammersmith Hospital, Imperial College Healthcare NHS Trust, London, UK; 104 Conquest Hospital, East Sussex NHS Trust, Sussex, UK; 105 Leipzig Heart Center, Leipzig, Germany; 106 University Clinic Dusseldorf, Dusseldorf, Germany

**Keywords:** Hypertension, renal denervation, ultrasound, blood pressure

## Abstract

Endovascular ultrasound renal denervation (uRDN) reduced blood pressure (BP) compared to sham at 2 months in patients with resistant hypertension in the multicenter, blinded, randomized, sham-controlled RADIANCE-HTN TRIO trial. This analysis evaluates longer-term outcomes of patients randomized to uRDN. Patients with resistant hypertension to a 3-drug combination pill were randomized to uRDN (*n* = 69) or sham (*n* = 67). From 2-5 months, patients followed a standardized anti-hypertensive medication (AHM) titration protocol. At 6 months, patients were unblinded and received AHM per standard of care. In the uRDN group, 71% (49/69) completed 36-month follow-up. Screening office BP was 159/103 on 3.9 AHM. Baseline office BP on the single-pill combination was 153/99 mmHg. At 36 months, office BP changed by −14.5 ± 26.1/−9.0 ± 14.8 mmHg from screening (*p* < 0.001 for both) and −8.0 ± 24.5/−5.0 ± 14.6 mmHg from baseline (*p* = 0.007; *p* = 0.022) on 3.7 AHM. The efficacy of uRDN was durable to 36 months in patients with resistant hypertension with no safety concerns.

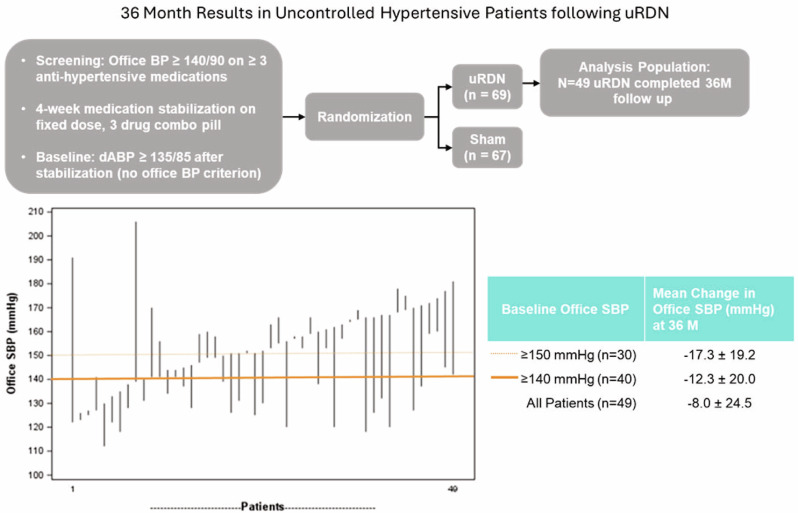

## Introduction

Endovascular ultrasound renal denervation (uRDN) effectively lowers daytime ambulatory systolic blood pressure in mild-to-moderate hypertension patients off anti-hypertensive medications (AHM) and those resistant to a single-pill fixed-dose of three AHMs [1]. While methodological and ethical considerations required the primary endpoint of uRDN studies to be at 2 months, the durability of the BP-lowering effect has been a topic of debate. This analysis evaluates the long-term efficacy and safety of uRDN over 36 months in resistant hypertensive patients under standard-of-care medications enrolled in the international, multicenter, randomized, sham-controlled RADIANCE-HTN TRIO trial, which was approved by local committees.

## Methods

In RADIANCE-HTN TRIO, patients with office BP ≥ 140/ 90 mmHg despite the use of ≥3 AHM at screening were switched to single-pill, fixed-dose combination of three medications (olmesartan/valsartan, amlodipine, hydrochlorothiazide) and then randomized to uRDN or sham if their daytime ambulatory BP was ≥135/85 mmHg, estimated glomerular filtration rate (eGFR) was ≥40 ml/min/1.73 m^2^, and they had suitable renal anatomy [1]. From 2 to 5 months, if monthly home BP was ≥135/85 mmHg, prespecified standardized stepped-care AHM was started under continuous blinding of patients and physicians [1]. After unblinding at 6-months, patients received AHM at the physician’s discretion according to local standard of care [2]. After the primary endpoint was met, unblinded crossover for patients initially randomized to sham was permitted if specific criteria were met.

## Results

In the uRDN group, 71% (49/69) completed their 36-month visit (mean age 53 years, 18% (9/49) female, 69% (34/49) White). Mean screening office BP was 159/103 mmHg on mean 3.9 AHM and mean baseline office BP was 153/99 mmHg on mean 3.1 AHM.

**Fig. 1:**
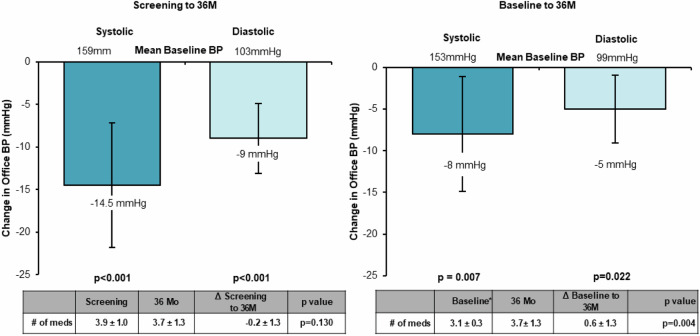
Change in Office Blood Pressure from screening (prior to standardization on triple pill) (Left) and change from baseline (after standardization on triple pill) (Right)

At 36 months, mean office BP in the uRDN group decreased by −14.5 ± 26.1/−9.0 ± 14.8 mmHg from screening (*p* < 0.001 for both) and -8.0 ± 24.5/-5.0 ± 14.6 mmHg from baseline (*p* = 0.007; *p* = 0.022) on a mean of 3.7 AHM (Fig. 1). A total of 17/49 (35%) patients had their office BP controlled (<140/90 mmHg) at 36 months.

**Fig. 2:**
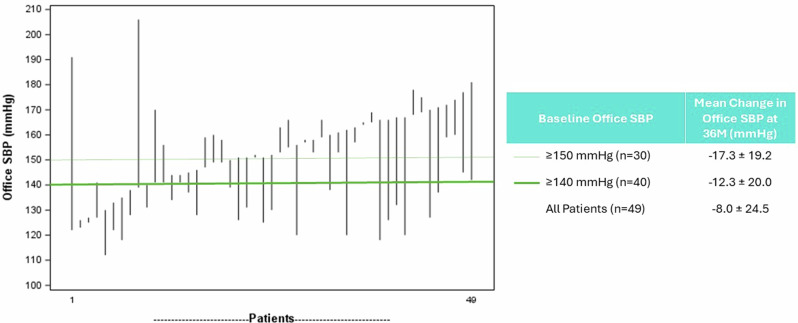
Individual Changes From Baseline to 36 Months in Office Systolic Blood Pressure - TRIO 36M Follow-up

The decrease in office BP varied by patient and was greater in those with higher baseline office BP (Fig. 2). Patients with uncontrolled BP at baseline (office systolic BP ≥ 140 mmHg) had a change of −12.3 ± 20.0 mmHg in office systolic BP at 36 months and patients with baseline office systolic BP ≥ 150 mmHg had a change of −17.3 ± 19.2 mmHg at 36 months. Patients with baseline office systolic BP ≥ 140 mmHg and ≥150 mmHg were on similar number of AHMs (3.6 and 3.9, respectively).

There was a small increase in AHM compared with baseline (+0.6 ± 1.3; *p* = 0.004), which may have impacted the difference in BP at 36 months. However, importantly, there was no increase in medication as compared with the initial screening visit (−0.2 ± 1.3; *p* = 0.130) suggesting a medication independent BP lowering effect. The majority of uRDN patients remained on a renin-angiotensin system blocker (94%; 46/49), a calcium channel blocker (88%; 43/49), and a thiazide-type diuretic (86%; 42/49) at 36 months; 76%; 37/49 were taking were taking all three of these drugs. Forty-one percent (20/49) were taking aldosterone antagonists. There were no new safety signals during the 36-month follow-up and no clinically significant (>50%) site-reported renal artery stenosis. There was one early death previously reported and unrelated to the device or procedure [2].

## Discussion

These findings enhance understanding of uRDN’s long-term effectiveness in real-world scenarios, where patients received standard of care anti-hypertensives prescribed by their doctors post-unblinding. The comparison to pre-randomization baseline is valuable, but represents unique study-specific conditions, with monitored adherence to a single pill fixed-dose combination, differing from typical clinical practice. In addition, several patients had a low baseline office BP, due to no office BP inclusion criteria at baseline. For these reasons, we feel that the comparison to screening is the best demonstration regarding the magnitude of the real-world efficacy of uRDN.

Although most patients (65%; 32/49) did not achieve office BP control due to probable clinical inertia and/or non-adherence to AHM, the clinical implications of this magnitude of office BP reduction are likely substantial. The change in office BP from baseline was ≥10 mmHg in 49% (24/49) and ≥5 mmHg in 59% (29/49) of patients. Studies have shown systolic office BP reductions of 5 and 10 mmHg are associated with reductions in cardiovascular events of 10% and 20%, respectively [3, 4].

This analysis had limitations. Only office BP measurements were available at the 36-month visit and follow-up rate was suboptimal. Unblinding to original treatment allocation after 6 months, unrestricted AHM prescription, and discontinuation of chemical adherence testing may have confounded results. Finally, there was no suitable sham group for comparison, due to crossover allowance post-primary endpoint. However, these factors may enhance the study’s real-world relevance.

In conclusion, these data reinforce the safety and durable effectiveness of uRDN up to 36 months in patients with resistant hypertension who remain on standard of care background medication.
